# A full-length transcriptome and gene expression analysis of three detoxification gene families in a predatory stink bug, *Picromerus lewisi*

**DOI:** 10.3389/fphys.2022.1016582

**Published:** 2022-10-10

**Authors:** Wenhong Li, Xinyi Wang, Po Jiang, Mingwei Yang, Zhimo Li, Chunyang Huang, Yueping He

**Affiliations:** ^1^ Institute of Plant Protection, Guizhou Academy of Agricultural Sciences, Guiyang, China; ^2^ Hubei Insect Resources Utilization and Sustainable Pest Management Key Laboratory, College of Plant Science and Technology, Huazhong Agricultural University, Wuhan, China; ^3^ Guizhou Provincial Tobacco Company, Zunyi Branch, Zunyi, China

**Keywords:** Picromerus lewisi, predatory stink bug, iso-seq, cytochrome P450 monooxygenase, glutathione S-transferase, carboxylesterase

## Abstract

The predatory stink bug *P. Lewisi* shows potential for Integrated Pest Management programs for controlling Lepidoptera pest insects in crops and forests. The importance of this insect for biological control has stimulated several studies into its biology and ecology. However, *P. lewisi* has little genetic information available. In the present study, PacBio single-molecule real-time (SMRT) sequencing and Illumina RNA-seq sequencing technologies were used to reveal the full-length transcriptome profiling and tissue-specific expression patterns of *P. lewisi*. A total of 12,997 high-quality transcripts with an average length of 2,292 bp were obtained from different stages of *P. lewisi* using SMRT sequencing. Among these, 12,101 were successfully annotated in seven public databases. A total of 67 genes of cytochrome P450 monooxygenases, 43 carboxylesterase genes, and 18 glutathione S-transferase genes were identified, most of which were obtained with full-length ORFs. Then, tissue-specific expression patterns of 5th instar nymphs were analyzed using Illumina sequencing. Several candidate genes related to detoxification of insecticides and other xenobiotics as well as the degradation of odors, were identified in the guts and antennae of *P. lewisi*. The current study offered in-depth knowledge to understand the biology and ecology of this beneficial predator and related species.

## 1 Introduction

*Picromerus lewisi* (Hemiptera: Pentatomidae) is a predatory stink bug widely distributed in China, Korea, Japan, and Russia (Lin et al., 2000). *P. lewisi* is an excellent biological control agent of major pest insects in crops and forests. It often preys on insect pests, such as Lepidoptera larvae (Mu et al., 2022). Biology and ecology, including taxonomy, morphology, reproduction, predator behavior, and feeding strategies of *P. lewisi*, have been studied (Tang et al., 2019; Wang et al., 2019; Wang et al., 2020; Mu et al., 2022). However, knowledge of the genomic and transcriptomic data is required to fully in-depth understand the biology and ecology of this cruicial pest.

Currently, *P. lewisi* has been mass-bred by factories and released to manage pest insects in China. Guizhou has the largest number of *P. lewisi* breeding centers in China, among which the breeding center in Zunyi City is one center with the largest capacity in China. However, more studies need to be conducted to improve its control efficiency. This predator might be exposed to insecticides in fields if used for pest control. Information on insecticide toxicities against predators and their mechanisms could help us to develop successful pest management strategies based on better compatibility between chemical and biological control methods. Information about the candidate genes related to insecticide detoxification allows us to investigate insecticide toxification mechanisms against the predatory bugs. A herbivory stink bug *Halyomorpha halys* was found to possess one of the highest number of detoxification genes, which might be due to its extreme generalist behavior (Bansal and Michel 2018; Sparks et al., 2020). The predatory stink bugs that exhibit omnivorous behavior and feed on plants and arthropods might have high detoxification adaptations and adopt more detoxifying enzymes to manage secondary toxic compounds or xenobiotics (Dumont et al., 2018). However, comprehensive investigation of detoxification genes in predatory stink bugs is still lacking.

Three key enzyme families comprising glutathione S-transferases (GSTs), Carboxyl/choline esterases (CCEs), and cytochrome P450 monooxygenases (CYPs) are linked with the degradation of insecticdes and other xenobiotics in insects (Oakeshott et al., 2010). CYPs comprise one of the largest gene families in insects. A large diversity of insect CYP genes are responsible for diverse adaptation to various habitats, selective pressures and physiological processes. Insect CYPs are distributed into four well-supported clades, CYP3, CYP4, CYP2, and mitochondrial CYP (CYPmito) clans; members of CYP3 clan are usually implicated in herbivore adaptation on xenobiotics (Feyereisen 2012). Insect CCEs are typically categorized into three classes: dietary/detoxification (DD), hormone and pheromone processing (HPP), and neuro/developmental (ND); DD and some HPP members are related to the detoxification of xenobiotics while ND members are non-catalytic proteins that have conserved functions on insect the nervous system (Oakeshott et al., 2010). Insect GSTs can be divided into seven classes: delta, epsilon, omega, sigma, theta, zeta, and microsomal; the expansion of delta and epsilon GST genes in insects are thought to be responsible for environmental adaptations and insecticide detoxification (Enayati et al., 2005).

Here, we performed the first full-length transcriptome analysis of *P. lewisi* using a third-generation single molecule-real time (SMRT) sequencing technology. Then the second-generation Illumina RNA-Seq sequencing technology was adopted to analyze the gene expression patterns of different tissues/parts of *P. lewisi*. In addition, we have thoroughly examined three detoxification gene families. The full-length transcripts and their tissue-specific expression patterns identified in this study will help to in-depth understand the functional genomics and adaptive evolution of this insect.

## 2 Materials and methods

### 2.1 Insects

*P. lewisi* was supplied by the natural enemy breeding center at Fenggang County of The Guizhou Tobacco Company Zunyi Branch, Zunyi, Guizhou Province, China. *Mythimna separata* larvae were used for rearing the bugs. The *M. separata* colony was reared on an artificial diet of corn leaf powder. The rearing condition for both *P. lewisi* and *M. separata* is held on 27 ± 1 °C, 75 ± 5% RH, and a 16:8 (L:D) h photoperiod.

### 2.2 SMRT sequencing

RNA samples with different insect stages were extracted from whole bodies of thirty 1^st^ instar nymphs, thirty second instar nymphs, twenty third instar nymphs, twenty fourth instar nymphs, ten 5th instar nymphs, ten female adults and ten male adults of *P. lewisi*, respectively, using TRIzol reagent (Invitrogen, United States) according to the manufacturer’s protocol. The mixed RNA sample with 3ug was used for PacBio SMRT sequencing. The mRNA was reverse-transcribed into cDNA using a SMARTer PCR cDNA Synthesis Kit (Takara Bio United States, Inc, CA, United States). The generated cDNA was then re-amplified using PCR. The Isoform Sequencing (Iso-Seq) library was prepared according to the Iso-Seq protocol using the SMRTbell™ Template Prep Kit (Pacific Biosciences, CA, United States). The qualified library was sequenced on a PacBio Sequel II platform using Sequel™ Sequencing Kit 2.0 (Pacific Biosciences, CA, United States).

The raw sequencing reads were processed using the SMRTlink 6.0 software (http://www.pacb.com/products-and-services/analyticalsofware/smrt-analysis/) to obtain sub-reads. Reads of insert (RoIs) were extracted from the subreads *via* self-correction. Then RoIs were classified into full-length non-chimeric (FLNC) reads, full-length chimeric reads, and non-full length reads, based on the identification of the 5′ and 3′ adapters used in the library preparation as well as the poly(A) tail. The FLNC reads containing 5′ and 3’ adapters and poly(A) tails were clustered to generate complete unigenes using the CD-HIT program (Fu et al., 2012). Raw data of the *P. lewisi* full-length transcriptome were available from the NCBI Short Read Archive (SRA) database (BioProject number: PRJNA863048).

All unigenes were *de novo* annotated against NR (National Center for Biotechnology Information [NCBI] non-redundant protein sequences), NT (NCBI nucleotide sequences), KEGG (Kyoto Encyclopedia of Genes and Genomes), Swiss-prot, GO (Gene Ontology), KOG (euKaryotic Ortholog Groups), and Pfam (Protein family) databases.

### 2.3 Illumina sequencing

Total RNA samples were extracted from different tissues or body parts of 100 fifth-instar nymphs, including guts (G), salivary glands (SG), antennae (A), legs (L), and heads without antennae and salivary glands (H). Nymphs of *P. lewisi* were starved for at least 6 h before extraction of RNA samples. Each tissue sample has three independent biological replicates. RNA amount and integrity were assessed using the RNA Nano 6000 Assay Kit in the Bioanalyzer 2,100 system (Agilent Technologies, United States). RNA sequencing (RNA-seq) was performed using illumina NovaSeq 6000 (illumina, United States) at Novogene Bioinformatics Technology Co., China. Each library was generated with 150 bp paired-end reads and approximately 20 million sequence reads.

Raw illumina data were filtered to remove reads containing adapter or poly-N, and low-quality reads through in-house perl script. The paired-end clean reads were mapped to the constructed Iso-Seq transcriptome database using bowtie2 (Langmead and Salzberg 2012). The raw reads of all RNA-seq libraries were deposited in the NCBI SRA (BioProject number: PRJNA862669).

### 2.4 Identification of metabolizing enzyme genes

CYP, CCE and GST genes were first screened *via* keyword search on the annotation table of *P. lewisi* transcriptome. The annotated detoxification protein sequences of two bugs, *H. halys* (Sparks et al., 2020) and *Rhodnius prolixus* (Traverso et al., 2017), were used for BLAST queries against the *P. lewisi* transcriptome to identify any missed genes. Subfamily identification for *P. lewisi* gene families was conducted using phylogenetic trees, based on the sequence alignment with annotated detoxification protein sequences from *H. halys* and *R. prolixus* by applying MEGA version 6 software (Tamura et al., 2013), using the maximum-likelihood method of the Jones-Taylor-Thornton (JTT) model and a bootstrap analysis with 1,000 replicates.

### 2.5 Differential expression analysis

Differential expression analysis was performed using the DESeq2 R 1.20.0 package (Love et al., 2014). The FPKM (fragments per kb per million fragments) method was used to calculate unigene expression (Trapnell et al., 2010). The resulting *p*-values (P_adj) were adjusted using Benjamini and Hochberg’s approach to control the false discovery rate. Differentially expressed genes (DEGs) were determined by setting | log2 (fold) | > 1 and adjusted *p*-value (P_adj) value <0.05. The heatmaps were constructed based on Log10 (FPKM +1) values among different tissues.

### 2.6 Quantitative real-time PCR validation of RNA-seq data

Eighteen genes were selected for data validation using qRT-PCR assay on an ABI Prism 7300 (Applied Biosystems, Foster City, CA) using SYBR Premix Ex Taq (Takara Biotechnology Corporation Co. Ltd., Dalian, China). Primers for qRT-PCR were presented in Supplemerntary Table S1. Three biological replicates and three technical replications were performed. The gene Pl_EF1A were selected for the candidate reference gene. The relative expression levels of the eighteen selected genes were calculated by the 2^−ΔΔCt^ method (Livak and Schmittgen 2001). The qRT-PCR data were statistically analyzed by one-way analysis of variance followed by Tukey’s multiple comparison test of significance using SPSS software (version 22.0, IBM Corp, Armonk, United States).

## 3 Results

### 3.1 Construction of a full-length transcriptome

A full-length transcriptome was sequenced from a mixed RNA sample of different stages of *P. lewisi* using SMRT technology. A total of 756,599 reads of inserts (RoIs) based on polymerase read fragment lengths >50 bp, a predicted consensus accuracy >0.8, and full passes >0 were obtained (Table 1). The mean read length of insert, mean read quality of insect and mean number of passes were 2,011 bp, 1.00, and 49.74, respectively (Table 1). In addition, 487,143 sequences (49.8%) were identified as full-length non-chimeric reads from RoIs. Based on the iterative clustering, polishing, and redundant removing, 12,997 high-quality unigenes with a mean length of 2,292 bp were generated (Table 1).

**TABLE 1:** Full-length transcriptomic analysis information.

Category	Number
Reads of inserts	756,599
Read bases of inserts	1,522,128,253
Mean read length of inserts	2,011
Mean read quality of inserts	1
Mean number of passes	49.74
Non-chimeric full-length reads	498,143
non full-length reads	225,723
Chimeric full-length reads	32,733
Total length (bp) of unigene	29,789,075
Unigene sequence number	12,997
Mean length (bp) of unigenes	2,291.99623
N50 (bp) of unigenes	2,876
GC% of unigenes	35.82

About 93.1 % of the 12,997 high-quality transcripts were annotated in at least one database. The blast alignment analysis showed that 85.5% of homologous transcripts were best matched to proteins from the herbivory stink bug *H. halys* (Supplementary Figure S1).

### 3.2 Construction of RNA-seq libraries

Through Illumina sequencing, 15 RNA-seq libraries were constructed from three repeats of five tissues or parts, G, H, A, L, and SG, of *P. lewisi* 5th-instar nymphs. More than 64.8 million reads in each sample were generated, resulting in the Q30 value higher than 92% in each sample after trimming and filtering (Supplementary File S1). To investigate the expression patterns of genes in *P. lewisi*, the Illumina clean reads were mapped to the SMRT full-length cDNA library. More than 78% of clean reads from each tissue library were mapped. A pearson correlation revealed distinct clustering of samples by tissue (Supplementary Figure S2). Pairwise comparisons among the five tissues were performed to identify the differentially expressed unigenes (DEGs). A smaller difference existed between guts and salivary glands (3,161 DEGs) and among heads, antennae, and legs (2,273∼2,652 DEGs) (SuplementaryTable S3).

### 3.3 Identification of genes encoding metabolizing enzymes

#### 3.3.1 CYPs

In the full-length transcriptome of *P. lewisi*, 67 transcripts were annotated as CYPs, 64 of which were full-length transcripts with open reading frames (ORF) (Supplementary Table S4). After the BLAST searches against NCBI databases, 42, 14, 5, and 6 transcripts were identified and grouped into CYP3, CYP4, CYP2, and CYPmito clans, respectively (Table 2). The number of CYP genes in *P. lewisi* was much higher than the other two predatory bugs, *Orius laevigatus* and *Cyrtorhinus lividipennis*, but less than those from *H. halys*, *R. prolixus*, and *Triatoma infestans* (Table 2).

**TABLE 2:** Numbers of CYP genes annotated in *Picromerus lewisi* and other bugs.

Clan	Predatory bugs			Hematophagous bugs		Herbivory bugs	
	*Picromerus lewisi*	*Orius laevigatus*	*Cyrtorhinus lividipennis*	*Rhodnius prolixus*	*Triatoma infestans*	*Halyomorpha halys*	*Murgantia histrionica*
CYP2	5	6	5	7	1	6	7
CYP3	42	34	27	55	65	87	43
CYP4	14	13	21	49	22	45	30
CYPmito	6	5	4	8	6	6	6
Total	67	58	57	119	94	141	86

Note: Numbers of CYP, genes annotated in other bugs were reported by Bailey et al. (2022) (*Orius laevigatus*), Liu et al. (2017) (*Cyrtorhinus lividipennis*), Traverso et al. (2017) (*Rhodnius prolixus* and *Triatoma infestans*), Sparks et al. (2020) (*Halyomorpha halys*) and Sparks et al. (2020) (*Murgantia histrionica*).

**FIGURE 1:**
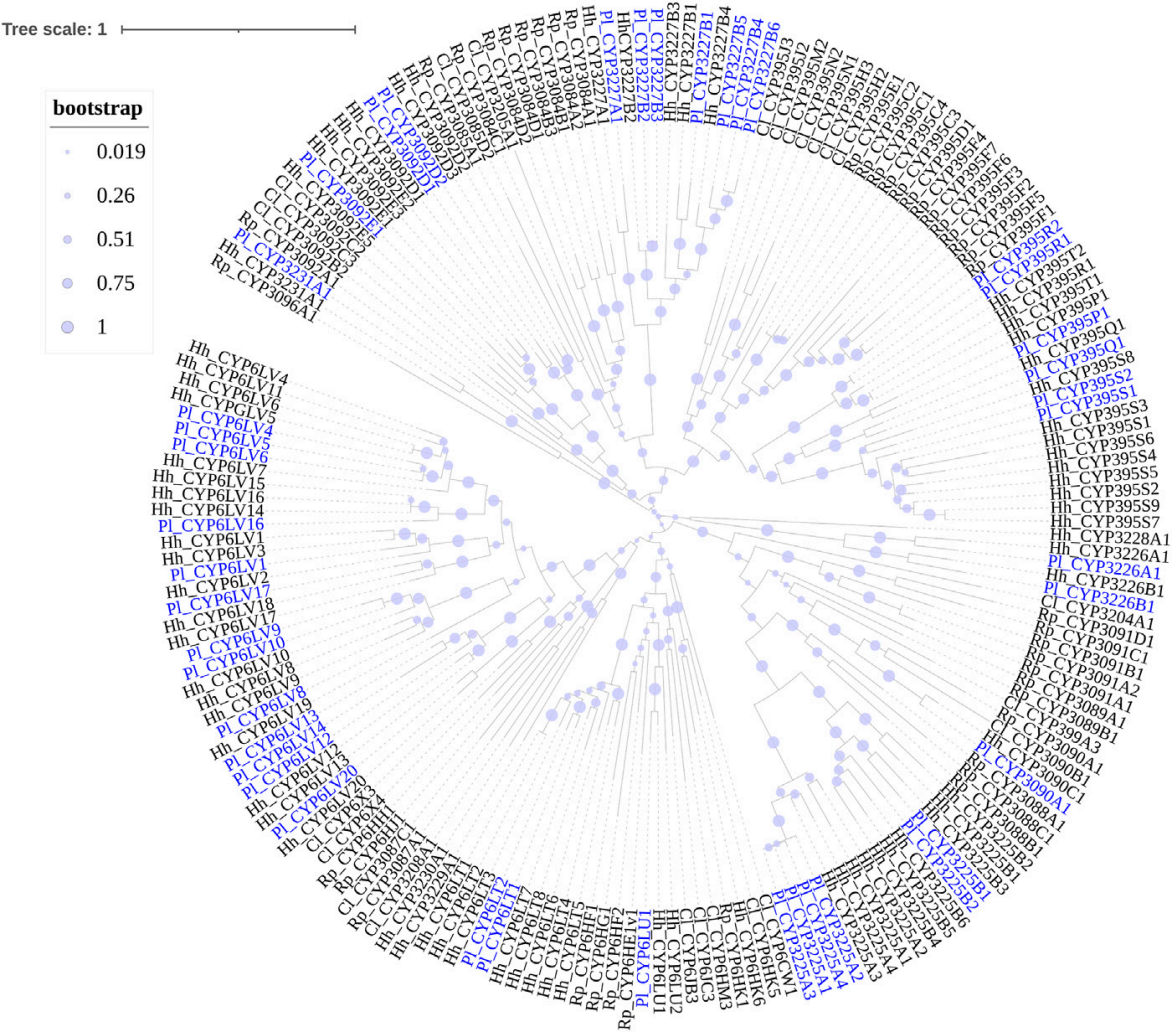
Phylogenetic tree of the CYP3 clan genes of *Picromerus lewisi* (Pl), *Halyomorpha halys* (Hl) (Sparks et al., 2020), *Rhodnius prolixus* (Rp) (Schama et al., 2016; Traverso et al., 2017) and *Cyrtorhinus lividipennis* (Cl) (Liu et al., 2017), constructed by the maximum likelihood method with the JTT model.

**FIGURE 2:**
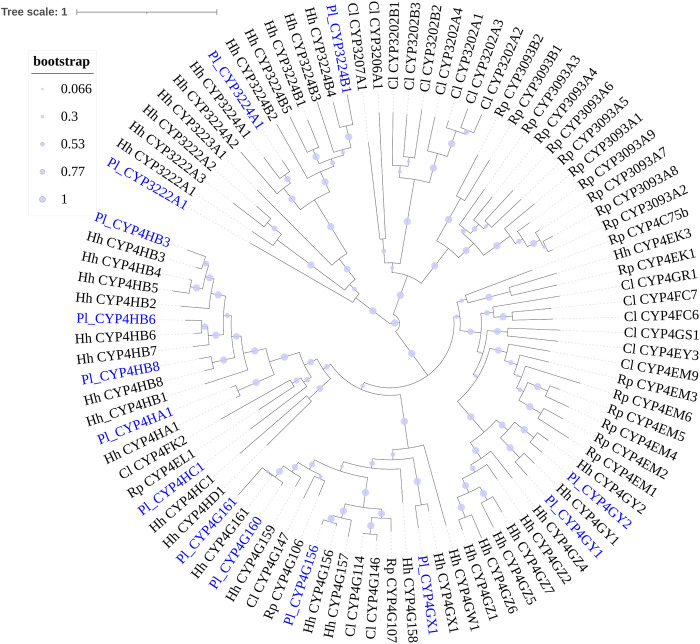
Phylogenetic tree of the CYP4 clan genes of *Picromerus lewisi* (Pl), *Halyomorpha halys* (Hl) (Sparks et al., 2020), *Rhodnius prolixus* (Rp) (Schama et al., 2016; Traverso et al., 2017) and *Cyrtorhinus lividipennis* (Cl) (Liu et al., 2017), constructed by the maximum likelihood method with the JTT model.

**FIGURE 3:**
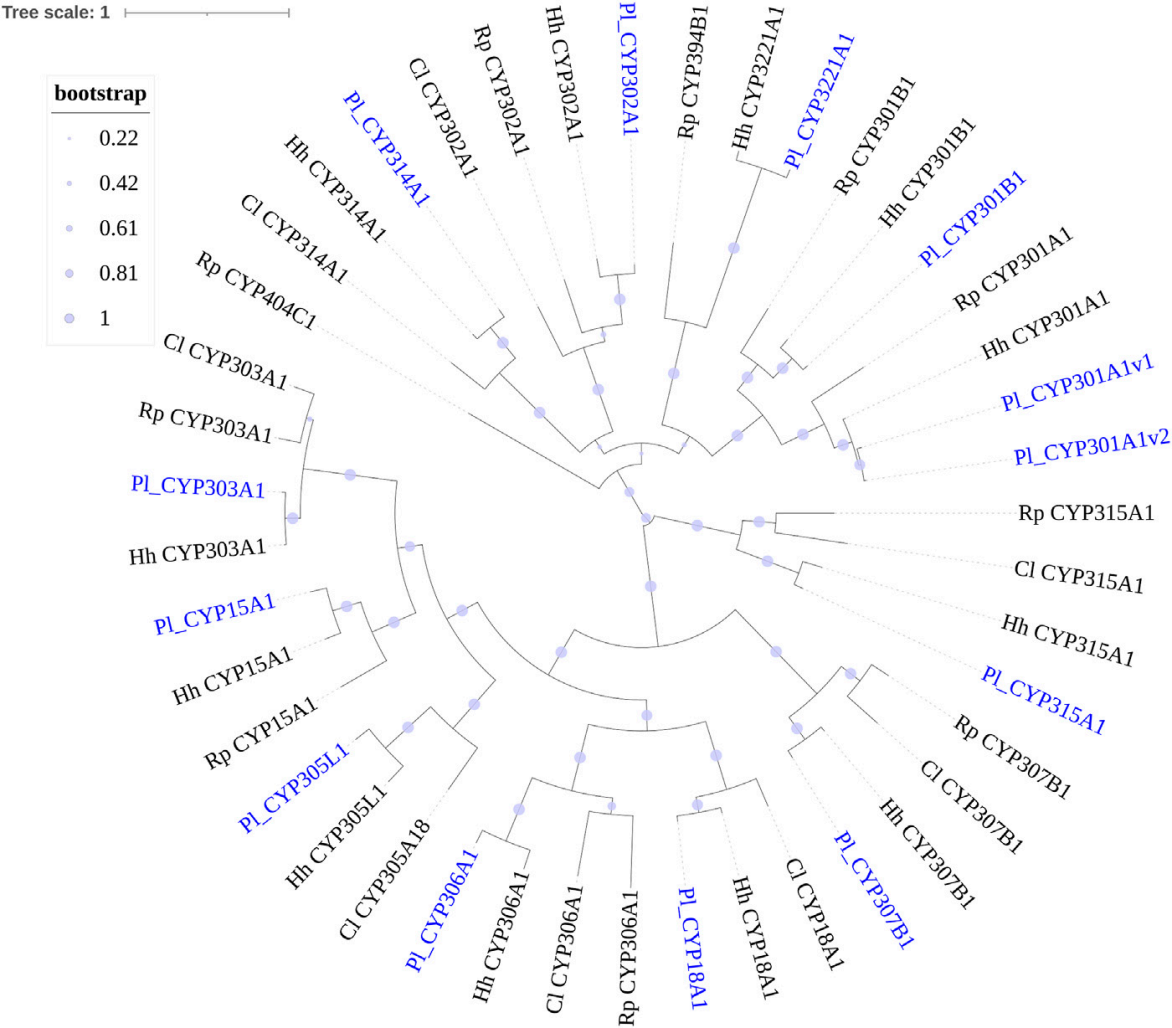
Phylogenetic tree of the CYP2 clan and CYPmito genes of *Picromerus lewisi* (Pl), *Halyomorpha halys* (Hl) (Sparks et al., 2020), *Rhodnius prolixus* (Rp) (Schama et al., 2016; Traverso et al., 2017) and *Cyrtorhinus lividipennis* (Cl) (Liu et al., 2017), constructed by the maximum likelihood method with the JTT model.

Phylogenetic trees were subsequently constructed to identify different CYP families, using the annotated CYP genes of *H. halys* (Sparks et al., 2020), *R. prolixus* (Schama et al., 2016), and *C. lividipennis* (Liu et al., 2017). According to phylogenetic relatedness to other named CYPs, all genes identified were assigned names. The CYP3 clan showed the largest expansion of P450s (42 genes) in the *P. lewisi* transcriptome, including 16 genes of three CYP6 families (CYP6LV, CYP6LT and CYP6LU) and 26 genes of 6 novel families (CYP395, CYP3092, CYP3225, CYP3226, CYP3227, and CY3231) (Table 3; Figure 1). CYP6LV (13/42) was the most abundant CYP3 family in *P. lewisi*. The CYP4 clan contained 11 CYP4 family genes and three genes of two novel families (CYP3222 and CYP3224) (Table 3; Figure 2). CYP3222 and CYP3224 were exclusive for pentatomids, while other new families of the CYP4 clan, such as CYP3093 expanded in *R. prolixus* are absent in *P. lewisi*. The CYP2 clan contained one gene for each CYP15, CYP18, CYP303, CYP306, and CYP307 (Table 3; Figure 3). Although CYP305 was not detected in the *P. lewisi* full-length transcriptome, it was found in the Illumina libraries (Figure 3). The mitochondrial clan contains two orthologs of CYP301A1 and one gene of each CYP301B1, CYP314A1, CYP315A1, and CYP3221 subfamilies (Table 3; Figure 3). CYP302 that was absent in the full-length transcriptome was identified in the Illumina libraries of *P. lewisi* (Figure 3).

**TABLE 3:** Tissue-specific expression patterns of CYP genes of *Picromerus lewisi via* RNA-seq.

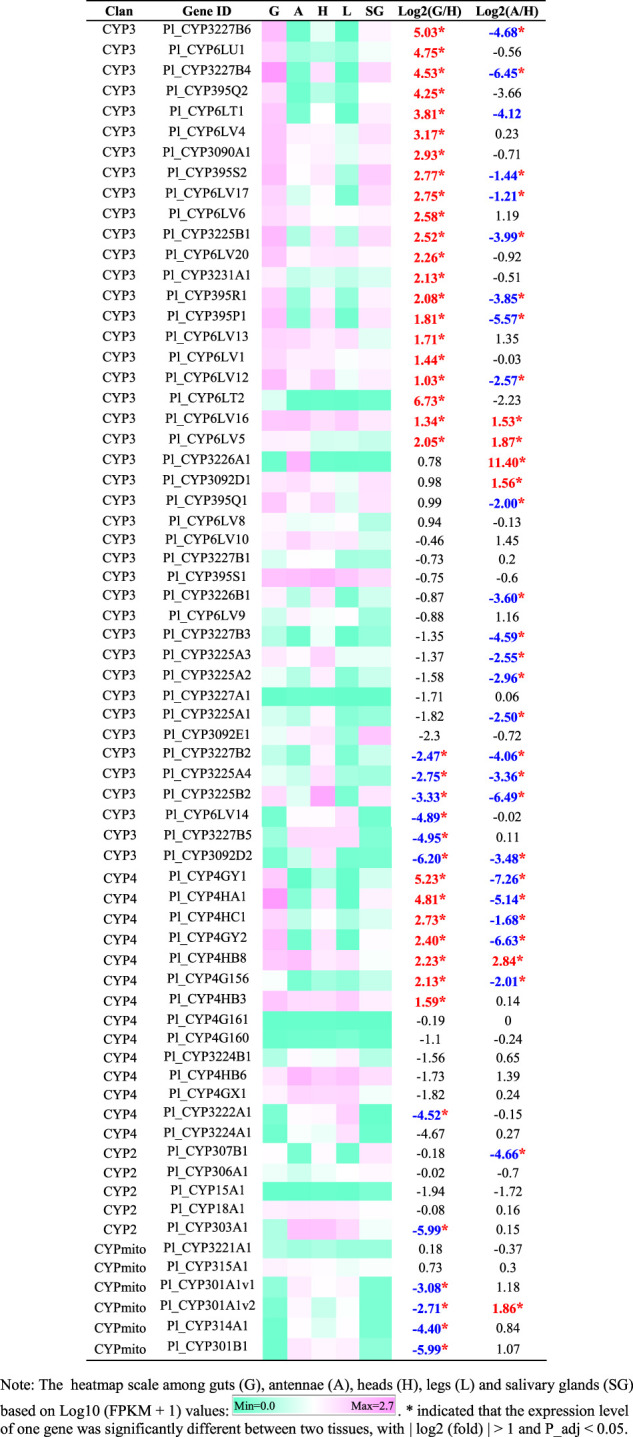

#### 3.3.2 CCEs

**FIGURE 4:**
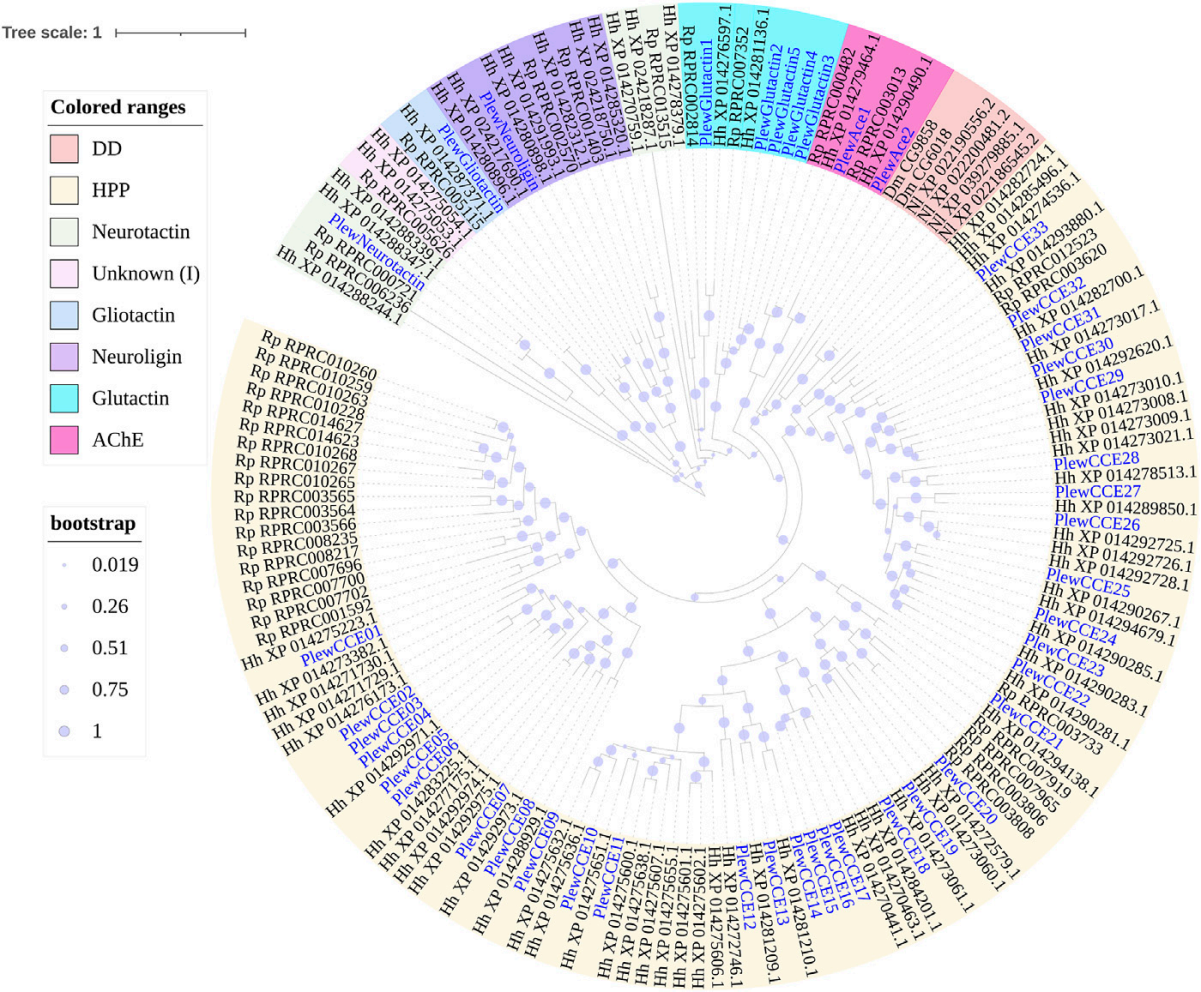
Phylogenetic tree of the CCE genes of *Picromerus lewisi* (Pl), *Halyomorpha halys* (Hl) (Sparks et al., 2020) and *Rhodnius prolixus* (Rp) (Schama et al., 2016; Traverso et al., 2017), constructed by the maximum likelihood method with the JTT model. Two *Drosophila melanogaster* (Dm) dietary/detoxification (DD) class genes and four *Nilaparvata lugens* (Nl) DD class genes were added into the tree, in order to showing the absent of DD class in Heteroptera. Expasion of the hormone and pheromone processing (HPP) class in bugs were shown in the tree.

A total of 43 putative CCE transcripts were found after annotating the full-length transcriptome, among which 36 transcripts with full length were identified (Table 4; Supplementary Table S4). Based on the BLAST searches against NCBI databases and the phylogeny tree analysis using the identified CCEs of *H. halys* (Sparks et al., 2020) and *R. prolixus* (Schama et al., 2016; Bailey et al., 2022), 33 and 10 CCEs of *P. lewisi* were identified and sorted into the HPP and ND classes, respectively (Table 5; Figure 4). DD class members were absent in *P. lewisi*. The ND class in *P. lewisi* contains two catalytic acetylcholinesterases (AChE-1 and AChE-2) and non-catalytic members such as glutactin, glioactin, neuroligin, and neurotactin (Table 5). The HPP class expanded in *P. lewisi* compared to the other predatory bugs, *O. laevigatus* and *C. lividipennis* (Table 4).

**TABLE 4:** Numbers of CCE genes annotated in *Picromerus lewisi* and other bugs.

s	*Picromerus lewisi*	*Orius laevigatus*	*Cyrtorhinus lividipennis*	*Rhodnius prolixus*	*Halyomorpha halys*
Detoxification/Dietary	0	0	0	0	0
Pheromone/hormone	33	16	12	40	55
Neuro/Developmental (total)	10	16	14	12	21
Clade H—Glutactin	5	1	2	2	2
Clade J—AChE	2	2	2	2	2
Clade K—Gliotactins	1	3	1	1	1
Clade L—Neuroligins	1	8	7	13	9
Clade M—Neurotactins	1	1	1	2	4
Unknown Function	0	1	1	1	3
Total CCEs	43	32	26	61	76*

Note: Numbers of CCE, genes in other bugs were reported by Bailey et al. (2022) (*Orius laevigatus*), Liu et al. (2017) (*Cyrtorhinus lividipennis*), Traverso et al. (2017) (*Rhodnius prolixus*) and Sparks et al. (2020) (*Halyomorpha halys*). *The number of CCE, genes in *H. halys* was revised to 76 after deleting 6 alternative splicing variants based on Sparks et al. (2020).

**TABLE 5:** Tissue-specific expression patterns of CCE genes of *Picromerus lewisi via* RNA-seq.

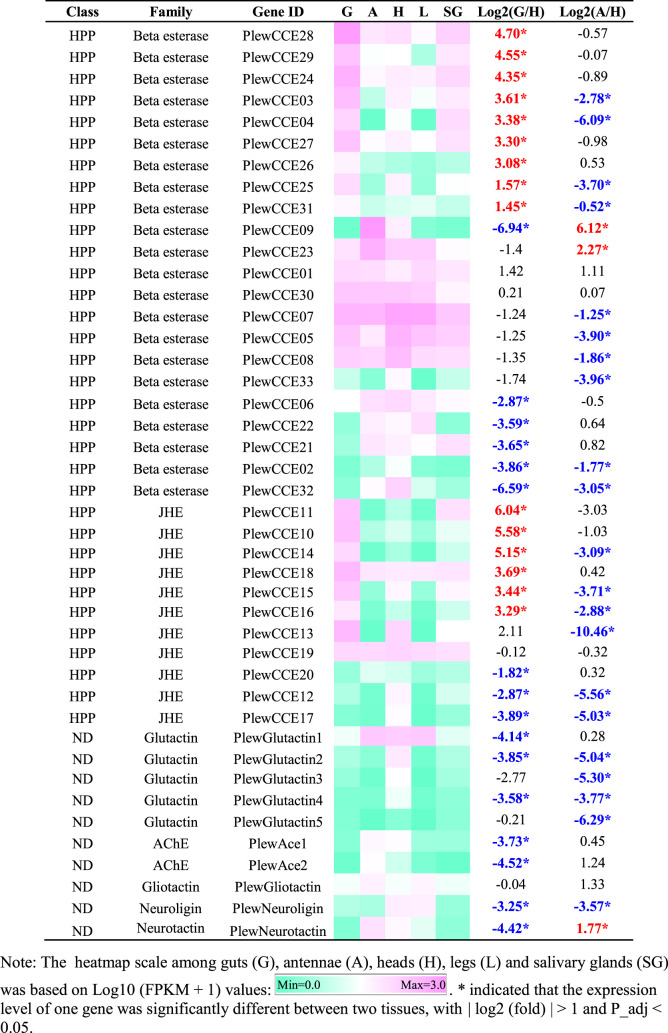

#### 3.3.3 GSTs

**FIGURE 5:**
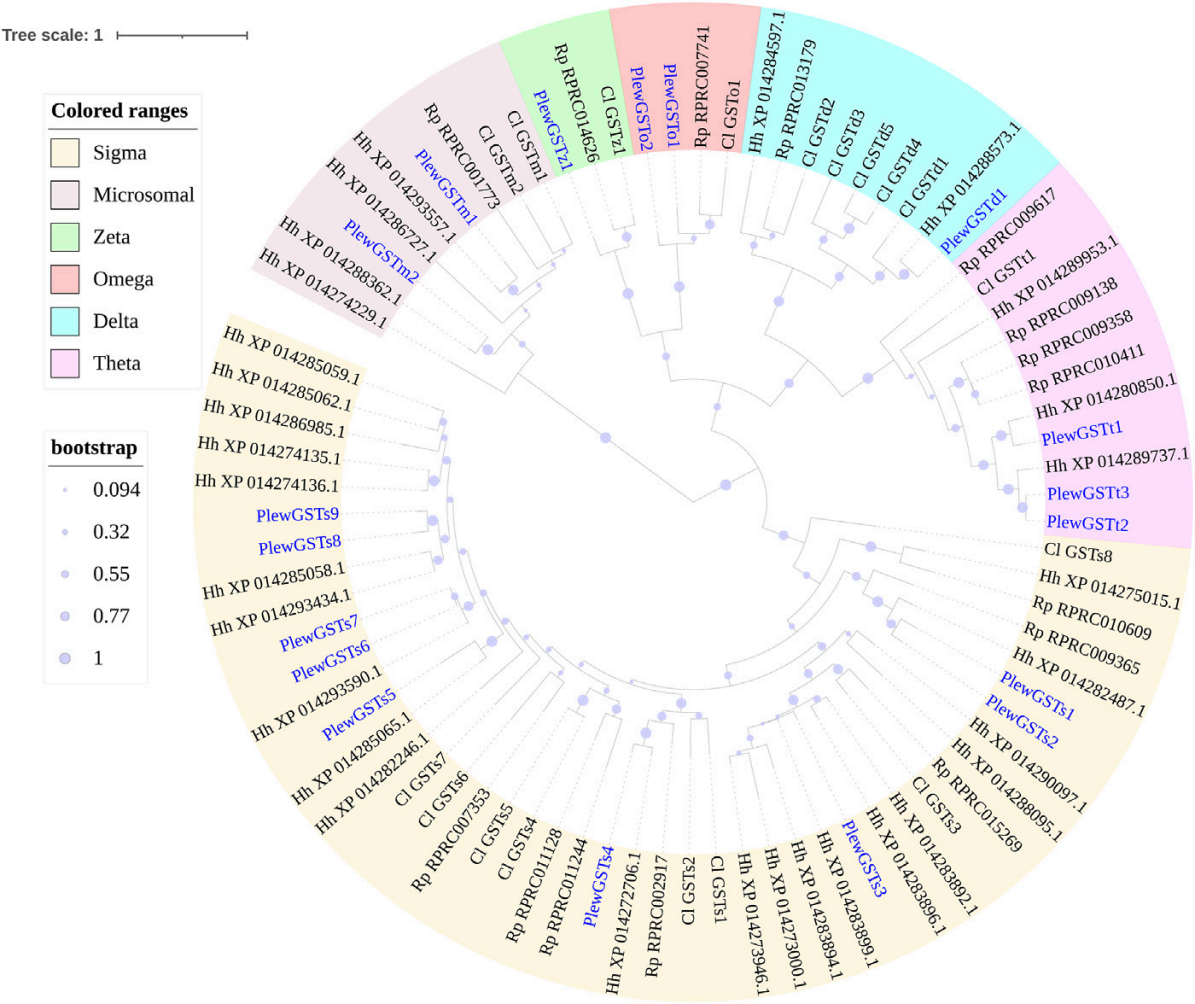
Phylogenetic tree of the GST genes of *Picromerus lewisi* (Pl), *Halyomorpha halys* (Hl) (Sparks et al., 2020), *Rhodnius prolixus* (Rp) (Schama et al., 2016; Traverso et al., 2017) and *Cyrtorhinus lividipennis* (Cl) (Liu et al., 2017), constructed by the maximum likelihood method with the JTT model. Expasion of the sigma class in bugs were shown in the tree.

In *P. lewisi*, 18 putative GST genes with full length were found (Table 6). Based on the phylogenetic analysis, six GST classes were identified in *P. lewisi*, including nine sigma members, three theta members, 2 Ω members, two microsomal members, one delta member, and one zeta member (Table 7; Figure 5). Epsilon GST genes were absent in *P. lewisi*. The sigma class was expanded to *P. lewisi* and other bug species (Table 6).

**TABLE 6:** Numbers of GST genes annotated in *Picromerus lewisi* and other bugs.

Class	*Picromerus lewisi*	*Orius laevigatus*	*Cyrtorhinus lividipennis*	*Rhodnius prolixus*	*Triatoma infestans*	*Halyomorpha halys*
Delta	1	1	5	1	1	2
Epsilon	0	0	0	0	0	0
Omega	2	2	1	1	0	3
Sigma	9	16	8	7	9	19
Theta	3	1	1	3	2	3
Zeta	1	1	1	1	0	1
Microsomal	2	3	2	1	2	5
Total	18	24	18	15	14	33

Note: Numbers of GST, genes in other bugs were reported by Bailey et al. (2022) (*Orius laevigatus*), Liu et al. (2017) (*Cyrtorhinus lividipennis*), Traverso et al. (2017) (*Rhodnius prolixus* and *Triatoma infestans*) and Sparks et al. (2020) (*Halyomorpha halys*).

**TABLE 7:** Tissue-specific expression patterns of GST genes of *Picromerus lewisi via* RNA-seq.

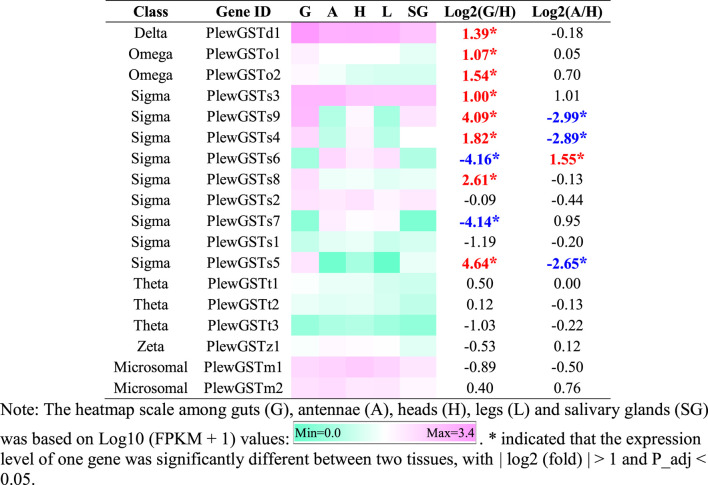

### 3.4 Tissue-specific expression profiles

Tissue-specific expression patterns from RNA-seq libraries by Illumina sequencing were analyzed to identify degrading genes enriched in guts and antennae of *P. lewisi*, which could be likely involved in xenobiotics detoxification and odorant processing.

A heatmap based on FPKM values of CYPs in guts, antennae, heads, legs, and salivary glands was shown in Table 3. Compared to heads, 21 CYP3 genes and 7 CYP4 genes were significantly upregulated in guts, with log2 (fold) > 1 and P_adj <0.05 (Table 3). In addition, six CYPs genes (*Pl_CYP3226A1*, *Pl_CYP6LV5*, *Pl_CYP3092D1*, *Pl_CYP6LV16*, *Pl_CYP4HB8* and *Pl_CYP301A1v2*) were significantly enriched in antennae, compared to heads (Table 3).

As shown in Table 5, 15 HPP CCE genes were significantly enriched in guts when compared to heads, whereas two HPP CCEs (*PlewCCE09* and *PlewCCE23*) and *PlewNeurotactin* were significantly upregulated in antennae, compared to heads (Table 5). Most ND class genes were enriched in heads (Table 5).

Amongst the 18 full-length GSTs, eight members exhibited higher expression levels in guts than heads, including one delta, 2 Ω, and five sigma members (Table 7). Only one GST gene (*PlewGSTs6*) was specifically enriched in antennae, compared to heads (Table 7).

**FIGURE 6:**
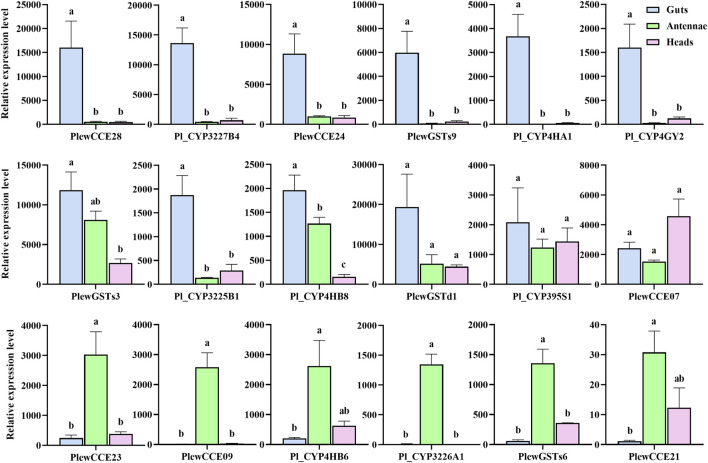
Relative expression levels of eighteen genes in guts, antennae and heads of *Picromerus lewisi* through qRT-PCR. Histograms represent relative expression ratios (means ± SE of three biological replicates), by setting the maximal one of all CT values to one using the 2^−ΔΔCt^ method. Different letters above histograms indicate statistically significant differences among different tissues/parts (*p* < 0.05).

To validate the DEGs identified through RNA-seq sequencing, eighteen genes were selected for qRT-PCR analysis (Figure 6). For all genes, the real-time PCR results were consistent with the expression profiles determined from RNA-Seq data. Among them, five CYPs (*Pl_CYP3227B4*, *Pl_CYP3225B1*, *Pl_CYP4HA1*, *Pl_CYP4GY2*, and *Pl_CYP4HB8*), two CCEs (*PlewCCE28* and *PlewCCE24*) and two GSTs (*PlewGSTs9* and *PlewGSTs3*) were significantly upregulated in guts, compared to heads. Five genes (*Pl_CYP4HB8*, *PlewCCE23*, *PlewCCE09*, *Pl_CYP3226A1* and *PlewGSTs6*) were enriched in antennae, compared to heads.

## 4 Discussion

SMRT sequencing can provide a new comprehension of full-length sequences, greatly facilitating the transcriptome research of species lacking high-quality reference genomes and improving genome annotation. To date, only a few full-length transcriptome analyses have been reported in insects, such as *Dendroctonus ponderosae* (Keeling et al., 2012), *Chouioia cunea* (Pan et al., 2019), *Sogatella furcifera* (Chen et al., 2020), *Aphis aurantii* (Hong et al., 2020), *Rhynchophorus ferrugineus* (Yang H et al., 2020), *Bactrocera dorsalis* (Ouyang et al., 2021), *Spodoptera frugiperda* (Yang et al., 2021), *Diabrotica virgifera* (Zhao et al., 2021). There are still no published reference genomes for predatory stink bugs. The present study provided a valuable genetic resource for further studies on the functional genomics and adaptive evolution of the predatory stink bugs and the close related herbivory stink bugs. To exhibit the advantages of gene discovery using SMRT sequencing, three major detoxification enzyme families were comprehensively investigated. A total of 67 CYPs, 43 CCEs, and 18 GSTs were identified from full-length transcriptome data of *P. lewisi*, most of which were obtained with full-length ORFs. Almost all of these genes of *P. lewisi* were grouped with their orthologues of *H. halys* in phylogenetic trees, supporting that predatory stink bugs evolved from the phytophagous stink bugs (Li et al., 2017; Liu et al., 2019).

Combined with Illumina sequencing, tissue-specific expression patterns of these metabolizing enzyme genes were analyzed to identify candidate degrading genes responding to xenobiotics and odorants. The insect gut is a key digestive organ that helps in the digestion and detoxification of xenobiotics and food. In *P. lewisi*, 30 CYPs, 15 CCEs, and eight GSTs exhibited significant upregulated expression levels in guts than heads, mainly expanded in the CYP3 clan and the CCE HPP class, supporting that most members of these classes were proposed to play important roles in xenobiotic detoxification.

Members of the CYP3 clan are usually implicated in herbivore adaptation to plant hosts and insecticides, mainly represented by CYP6 and CYP9 families (Feyereisen 2012). CYP9 members were absent in *P. lewisi*, consistent with the lack of CYP9 class in Hemiptera (Bailey et al., 2022). CYP6 is the most abundant CYP3 family in *P. lewisi*, among which subfamily CYP6LV is specificially expanded in *P. lewisi* and *H. halys* (Sparks et al., 2020). We observed that 50% (21/42) of CYP3 genes, 75% (12/16) of CYP6 genes and 69.2% (9/13) of CYP6LV genes of *P. lewisi* were enriched in the guts, compared to heads. Our observation suggested that members of the CYP3 clans represented by CYP6LV subfamily were the primary detoxifying gene group in the predatory stink bugs.

Some CYP4 members in insects were also reported to be involved in insecticide resistance and detoxification (Dulbecco et al., 2022). Our observation that 50% (7/14) of CYP4 genes, 80% (4/5) of CYP4H genes and 50% (3/6) of CYP4G genes in *P. lewisi* were upregulated in the guts, compared to heads, indicated that many CYP4 genes represented by CYP4H and CYP4G subfamilies might also be related to xenobiotic detoxification in *P. lewisi*.

Many genes belonging to the so-called dietary/detoxification (DD) class of CCEs have been have been involved in dietary/detoxification function. However, DD class members were absent in *P. lewisi*, consistent with recent studies in hematophagous bugs, predatory *O. laevigatus*, and herbivory Pentatomidae bugs (Bailey et al., 2022). In pentatomids, the HPP class harbors large expansions, which might have a role in detoxification. Our observation that about 50% (15/33) of HPP genes in *P. lewisi* were upregulated in the guts, compared to heads, supported such hypothesis.

In insects, delta and epsilon classes of GSTs were reported to be insecticide resistance and detoxification (Enayati et al., 2005). Epsilon genes were absent in *P. lewisi*, which is consistent with studies in other hemipteran species (Friedman 2011; Bailey et al., 2022). Only one delta gene was found in *P. lewisi*, with the highest expression level in guts among all of detoxification genes (Figure 6), indicating that this gene might be very important in detoxification function of the predatory stink bug. The expansion of the sigma class was found in *P. lewisi*, which is consistent with other studies reported in several hemipteran species, including *T. infestans*, *Myzus persicae*, *H. halys*, *Murgantia histrionica*, and *O. laevigatus* (Bailey et al., 2022). The sigma class has also been proved to be involved in the detoxification of insecticides in hemipteran and other species (Yamamoto et al., 2007; Gawande et al., 2014; Balakrishnan et al., 2019; Hassan et al., 2019; Yang Y et al., 2020; Liu et al., 2022). Our observation that 55.6% (5/9) of sigma GST genes in *P. lewisi* were upregulated in the guts, compared to heads, suggested that the sigma class of GSTs might also be related to xenobiotic detoxification in *P. lewisi*.

Some researchers hypothesized that these zoophytophagous predators should be more resistant to insecticides than strict predators, which might improve their ecosystem services in IPM programs (Dumont et al., 2018). *P. lewisi*, *O. laevigatus*, and *C. lividipennis* are zoophytophagous predators that sometimes feed plants. To adapt to new environments and consume more prey species, *P. lewisi* may require more detoxifying genes. Therefore *P. lewisi* contained more detoxification genes than *O. laevigatus* and *C. lividipennis*. However, several studies proved that some insecticides exhibited high toxicities or sub-lethal effects against predatory stink bugs (de Castro et al., 2014; Martinez et al., 2018; Santos Junior et al., 2020; Silva et al., 2020; Batista et al., 2022). The bugs might adopt different sets of detoxification genes to cope with insecticides and other xenobiotics from host plants and preys. Therefore the comprehensive study of insecticide toxification and its mechanism in *P. lewisi* needs to be further investigated, which will help improve its bio-control efficiency based on IPM strategies.

In the present study, several detoxification genes were identified to be enriched in antennae of *P. lewisi*, indicating that they might be odorant-degrading enzyme (ODE) genes. ODEs are a key player in insect olfaction dynamics. They play pivotal roles in the inactive metabolism of exogenous odorants and in the recovery of sensitivity in the olfactory system to detect new odorants (Chertemps and Maïbèche 2021). CYPs, CCEs and GSTs are known as three major ODE families in insects. To date, many detoxification enzymes involved in odorant degradation in insects have been identified, such as ApolPDE in *Antheraea polyphemus* (Vogt and Riddiford 1981), EST-6 in *Drosophila melanogaster* (Mane et al., 1983; Chertemps et al., 2015), SlitCXE7 and SlitCXE10 in *S. littoralis* (Durand et al., 2010; Durand et al., 2011), SexiCXE4 and SexiCXE14 in *S. exigua* (He et al., 2014; He et al., 2015), CYP345E2 in *Dendroctonus ponderosae* (Keeling et al., 2013), CYP4L4 in *S. litura* (Feng et al., 2017), GST-msolf1 in *Manduca sexta* (Rogers et al., 1999), GST-pxcs1 in *Papilio xuthus* (Ono et al., 2005), GmolGSTD1 in *Grapholita molesta* (Li et al., 2018), and SzeaGSTd1 in *Sitophilus zeamais* (Xia et al., 2022). The predatory stink bugs depend on their antennae to perceive a diversity of airborne chemical cues, including prey odorants, plant volatiles, sex pheromones, and scents from stink bugs, to find palatable preys, preferable host plants, and conspecific partners, and to avoid interspecific competition and natural enemies. These insects respond quickly to environmental cues, mainly relying on the termination of odorant signals from the olfactory sensilla (Chertemps and Maïbèche 2021). Therefore, identifying potential odorant-degrading genes in the predatory stink bugs may assist future research on their olfactory functions and the enhancement of predatory biocontrol efficiency.

Besides detoxification and odor degradation, insect CYP members in other insect species are also involved in the metabolism of endogenous compounds such as lipids, ecdysteroids and pheromones, and in the last step of cuticular hydrocarbon biosynthesis (Qiu et al., 2012). However, further studies are required to confirm that increased CYP genes in stink bugs might be involved in the synthesis or metabolism of specific stink smell chemicals (Sparks et al., 2020).

In addition, CYP6BQ9 was mostly expressed in *Tribolium castaneum* brains and conferred deltamethrin resistance, suggesting that CYPs specifically expressed in insect heads probably have detoxifying functions (Zhu et al., 2010). The central nervous system, particularly the brain, should be considered a target tissue to uncover more insect P450s involved in insecticide resistance because most insecticides are neurotoxins (Zhu et al., 2010). Several CYPs and other metabolizing genes of *P. lewisi* were overexpressed in the head, which needs further studies to investigate their functions.

## 5 Conclusion

This is the first report of a comprehensive characterization of the global transcriptome of *P. lewisi*. In the current study, the sequencing strategy combined PacBio SMRT with Illumina RNA-Seq was proved to exhibit good performance for gene identification, using three detoxification enzyme superfamlies as an example. A total of 67 genes of CYPs, 43 CCEs, and 18 GSTs were identified, most of which were obtained with full-length ORFs. More than 50 candidate xenobiotic detoxification genes enriched in the guts of *P. lewisi* and several candidate ODE genes upregulated in the antennae were identified based on tissue-specific transcriptomic analysis. The results presented here provide basic information for studying the predatory stink bugs’ adaptation mechanism to environmental factors.

## Data Availability

The datasets presented in this study can be found in online repositories. The names of the repository/repositories and accession number(s) can be found in the article/Supplementary Material.
