# Supplementary material for: A full-length transcriptome and gene expression analysis of three detoxification gene families in a predatory stink bug, Picromerus lewisi
Source: Front Physiol. 2022 Oct 10;13:1016582. doi: 10.3389/fphys.2022.1016582 (PMC9589283; doi:10.3389/fphys.2022.1016582)
Supplement: Supplementary file 1 [file DataSheet1.docx]

Supplementary Material

# Supplementary Figures and Tables

## Supplementary Tables

Table S1 Primers used in this study

| **Primer name** | **Primer sequence** |
| --- | --- |
| Pl_CYP3227B4-F | CGATACTTATGCTCCTGG |
| Pl_CYP3227B4-R | GCCATGGTTTGAAGTGCT |
| Pl_CYP3225B1-F | CGTTACTCTGTGCCTGGT |
| Pl_CYP3225B1-R | CCGCCTGTCCAAATATTA |
| Pl_CYP3226A1-F | GTTGGGTCTGATTGCATCAT |
| Pl_CYP3226A1-R | CATGTTACCTACGACCATCT |
| Pl_CYP395S1-F | GCTGTCCTCATTTTAGCC |
| Pl_CYP395S1-R | AATGGCCAATGACGGGAA |
| Pl_CYP4HA1-F | CCAGTGTTGATCGCCCTTAC |
| Pl_CYP4HA1-R | GTTTCCGACGATGGGAGAGG |
| Pl_CYP4HB6-F | ACCAAAGGGAGCCCATCTCGATATA |
| Pl_CYP4HB6-R | CCAGCCGAGAATGGTATGTATGCAT |
| Pl_CYP4HB8-F | CTGGATGTTTTCTTCCCC |
| Pl_CYP4HB8-R | CAACACTTTGGGGCATTC |
| Pl_CYP4GY2-F | CGTGGTTTTGTACGCTGT |
| Pl_CYP4GY2-R | GGATTAGAGCCAACTCCA |
| PlewCCE07-F | GCAGGGAGTGTCTATCATCACATCG |
| PlewCCE07-R | AGATTACGGTAGAGTCGATCTGCC |
| PlewCCE09-F | GGCCCATCGTTGAAATCTCT |
| PlewCCE09-R | ATCCTTCTGCTGCTTCTGGA |
| PlewCCE21-F | TGGCTCTGGATCTTCCTCCATGTAT |
| PlewCCE21-R | GTCCAGTGTAAAGCTGCTAACTGG |
| PlewCCE23-F | GCCGATAGGAGAGTACAG |
| PlewCCE23-R | GCAGTCTTCTGATCCGTG |
| PlewCCE24-F | GTGTAAGCCACGGAGATG |
| PlewCCE24-R | CCCAGTTCTCTCCTAACG |
| PlewCCE28-F | GGTGGATATGTGGGTCTC |
| PlewCCE28-R | GTTGTCTTAGCGGAAGGG |
| PlewGSTd1-F | CGATTTCTACTACGTTCCGG |
| PlewGSTd1-R | GGGTCATGTGTTCTCCTTTC |
| PlewGSTs3-F | ACCAGTCGACTGCTATCTCTCGTTA |
| PlewGSTs3-R | CATATCATGGAGTGTGTCGACAGCA |
| PlewGSTs6-F | GCGTTACTCCTGAAGAATGG |
| PlewGSTs6-R | GCGTTTTTACCAGCTAAGCC |
| PlewGSTs9-F | GTGTTACGCAAGAAGAATGG |
| PlewGSTs9-R | CTCAGCATTATCTCCAGCTA |
| Pl_EF1A-F | ACTGGTGTGCTTAAACCAGGTATGG |
| Pl_EF1A-R | GTGGTTCAATACGATGACCTGAGC |

Table S2 RNA-seq transcriptomic analysis information

| **Sample** | **Raw_reads** | **Raw_base** | **Clean_reads** | **Clean_bases** | **Error_rate** | **Q20** | **Q30** | **GC_pct** |
| --- | --- | --- | --- | --- | --- | --- | --- | --- |
| L1 | 23165693 | 6.95G | 22578453 | 6.77G | 0.03 | 97.13 | 92.48 | 44.34 |
| L2 | 22884026 | 6.87G | 22118767 | 6.64G | 0.03 | 97.33 | 92.88 | 44.93 |
| L3 | 22008882 | 6.6G | 21124400 | 6.34G | 0.03 | 97.21 | 92.76 | 46.07 |
| A1 | 22852923 | 6.86G | 22077298 | 6.62G | 0.03 | 97.24 | 92.63 | 40.34 |
| A2 | 22020726 | 6.61G | 21151782 | 6.35G | 0.03 | 97.16 | 92.55 | 40.55 |
| A3 | 22582403 | 6.77G | 21285897 | 6.39G | 0.03 | 97.09 | 92.39 | 38.93 |
| H1 | 20683396 | 6.21G | 19503916 | 5.85G | 0.03 | 97.4 | 93.06 | 41.46 |
| H2 | 21738729 | 6.52G | 20775311 | 6.23G | 0.03 | 97.28 | 92.72 | 39.49 |
| H3 | 23120928 | 6.94G | 22049663 | 6.61G | 0.03 | 97.31 | 92.78 | 42.04 |
| G1 | 21963061 | 6.59G | 20871296 | 6.26G | 0.03 | 97.41 | 93.01 | 40.8 |
| G2 | 23483358 | 7.05G | 22262170 | 6.68G | 0.03 | 97.42 | 92.91 | 40.94 |
| G3 | 22801099 | 6.84G | 21653893 | 6.5G | 0.03 | 97.49 | 93.11 | 41.58 |
| SG1 | 23461192 | 7.04G | 22319172 | 6.7G | 0.03 | 97.41 | 92.9 | 43.8 |
| SG2 | 22704739 | 6.81G | 21609314 | 6.48G | 0.03 | 97.41 | 92.95 | 43.19 |
| SG3 | 21587943 | 6.48G | 20341095 | 6.1G | 0.03 | 97.28 | 92.8 | 42.82 |

Table S3 The numbers of different expressed transcripts in different samples of RNA-seq transcriptomes. (| log2 (fold) | > 1 and P_adj value < 0.05)

| **Compare** | **All** | **Up** | **Down** |
| --- | --- | --- | --- |
| LvsSG | 4372 | 2412 | 1960 |
| AvsSG | 4705 | 2371 | 2334 |
| HvsSG | 4375 | 2735 | 1640 |
| GvsSG | 3161 | 1702 | 1459 |
| AvsH | 2652 | 672 | 1980 |
| LvsH | 2273 | 794 | 1479 |
| LvsA | 2522 | 1409 | 1113 |
| GvsH | 4903 | 1994 | 2909 |

## Supplementary Figures

**Figure S1** Similarity distribution (A) and species distribution (B) of unigenes of full-length trancriptome that hit in the NCBI NR protein database


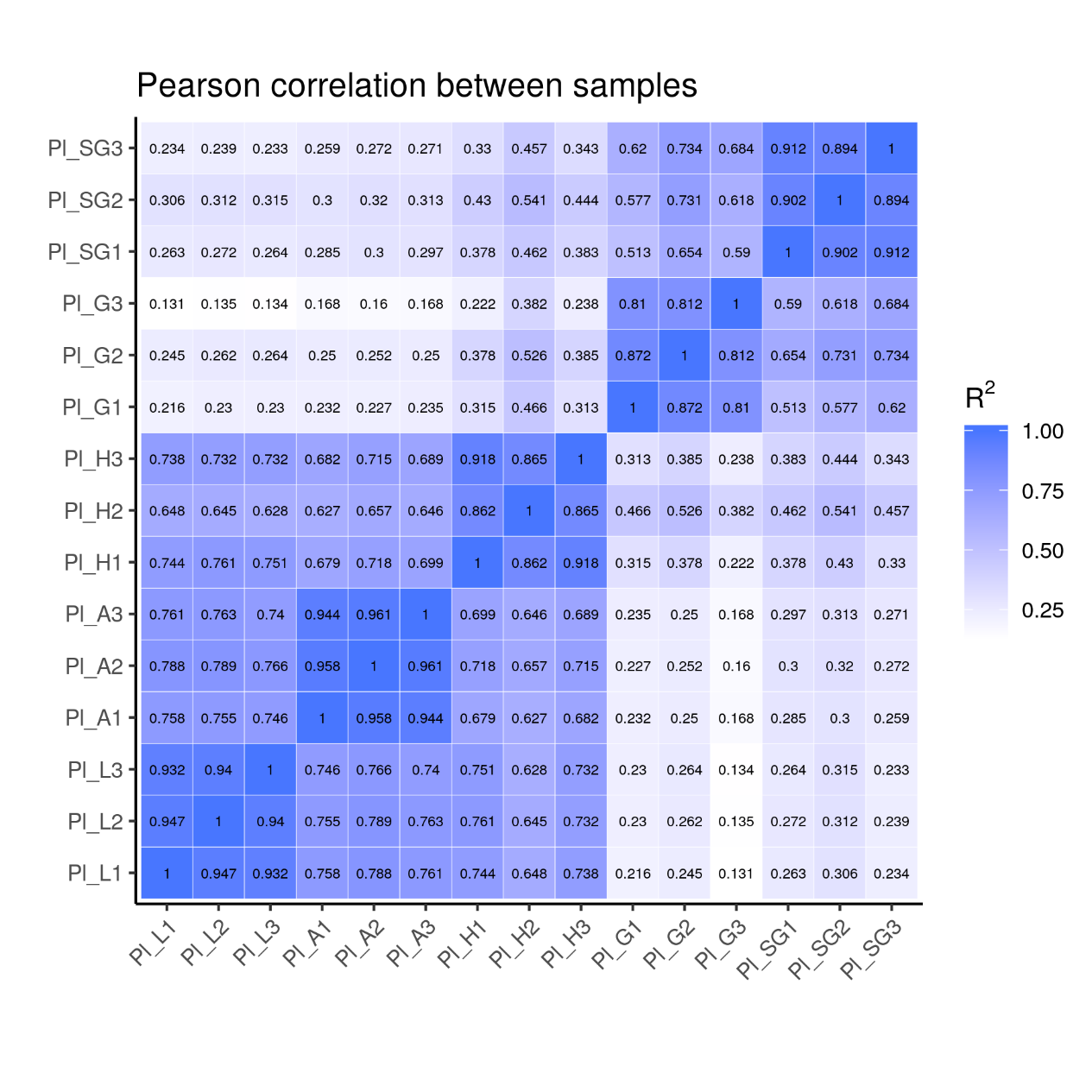


**Figure S2** Pearson correlation analysis of RNA-seq transcriptomic data.
